# How can the left atrial strain be correctly determined by cardiovascular magnetic resonance feature tracking?

**DOI:** 10.3389/fcvm.2025.1558102

**Published:** 2025-03-31

**Authors:** Rehsan Akkuzu, Hermann Körperich, Niroshan Shanmugarajah, Tobias Rossnagel, Oliver M. Weber, Christian Stehning, Wolfgang Burchert, Jan Eckstein

**Affiliations:** ^1^ Institute for Radiology, Nuclear Medicine and Molecular Imaging, Heart and Diabetes Center North-Rhine Westphalia, Ruhr-University of Bochum, Bad Oeynhausen, Germany; ^2^ Philips Clinical Science, Hamburg, Germany

**Keywords:** feature tracking, volumetrics, planning procedures, cardiovascular magnetic resonance, left atrial strain

## Abstract

**Introduction:**

Left atrial (LA) strain marks a highly valuable clinical parameter for discrimination of various cardiovascular diseases as consolidated by a plethora of literature. However LA strain assessment by cardiovascular magnetic resonance feature tracking (CMR-FT) is conventionally based on 2-chamber (2Ch) and 4-chamber (4Ch) cine acquisitions, which are aligned for left ventricular (LV) imaging. These slice positions are hypothesized inadequate for identifying the longest LA dimensions required for accurate LA strain quantification.

**Materials and methods:**

LA strain was assessed in 21 patients (48.3 ± 15.2 years, 10 females) with various cardiovascular diseases by CMR-FT. Two different planning procedures were compared: (1) the standard planning procedure using the cine steady-state free-precession (SSFP) 2Ch and 4Ch views aligned to the LV and (2) the optimized planning procedure based on cine SSFP 2Ch and 4Ch views, however, aligned to the LA. Strain analysis was performed using CVI42® software. Paired Student's *t*-test or Wilcoxon test and Bland Altman statistics were used to evaluate differences between both planning procedures.

**Results:**

Indexed maximum, minimum LA volumes and LA volumes before atrial contraction were significantly elevated for optimized planning compared to standard planning (*P* < 0.001). In contrast, global longitudinal reservoir, conduit and booster LA strain and consecutively strain rates were found reduced for the optimized planning procedure compared to the standard planning procedure (*P* < 0.007). The total and passive LA ejection fractions remained unchanged, whereas the booster LA ejection fractions was significantly lower for the optimized planning procedure (*P* = 0.034).

**Conclusion:**

Optimized LA planning procedure for assessment of the longest LA dimensions results in significant alterations in CMR quantifications with increased chamber volumetrics and decreased strain and strain rates compared to standard procedure.

## Introduction

Accurately assessing left atrial (LA) volume is clinically essential, as it serves as a crucial biomarker for cardiovascular health, particularly in conditions such as heart failure (1) and cardiac amyloidosis (2). Moreover LA enlargement is a well-established predictor of adverse cardiovascular outcomes, including atrial fibrillation (3), heart failure (4), and mortality (5). Since LA remodeling reflects chronic pressure and volume overload, precise quantification provides valuable insights into disease progression and the effectiveness of therapeutic interventions. Although LA strain is routinely assessed using echocardiography, this method has notable limitations, such as dependency on operator experience and patient echogenicity (6). Overcoming these limitations, cardiovascular magnetic resonance imaging (CMR) has become the established gold standard for cardiac functional analysis, offering independence from examiner competence, high reproducibility and excellent spatial and temporal resolution (7).

Despite the wealth of literature available on the clinical and prognostic value of CMR LA strain (8–10), a critical technical aspect of LA strain and volumetric quantification has remained neglected. The standard CMR planning technique is primarily designed for left ventricular strain analysis, which can lead to incomplete capture of the left atrium geometrics, underestimation of the longest LA extension and deviation of its centerline. The need for precise alignment of the highest point on the cranial aspect of the left atrium, coupled with the challenges posed by minimal alignment discrepancies, can result in varying outcomes. Consequently, LA strain and volumetrics may diverge significantly from their “true” values due to incomplete representation of all atrial segments.

This study aims to comprehensively evaluate the differences in LA volumetrics and strain when comparing optimized vs. standard CMR planning techniques. It is hypothesized that significant discrepancies in LA volumetric and strain values between the two CMR planning methods exist.

## Methods

For the purpose of this study 23 patients were initially recruited. The local ethics committee approved the study conditions (Ethikkommission der Medizinischen Fakultät der Ruhr-Universität Bochum, Sitz Bad Oeynhausen, registration number: 2017-238_4). All examinations were done in accordance with the 1964 declaration of Helsinki. A written informed consent was obtained from all participants. One patient was excluded due to metallic artifacts and one patient due to severe susceptibility artifacts. The final study group thus comprised 21 patients with different cardiovascular diseases (5 myocarditis, 3 hypertrophic cardiomyopathy, 2 non-compaction cardiomyopathy, 3 dilatative cardiomyopathy, 1 ectasia of ascending aorta, 1 patient with pectus excavatum, 1 sarcoidosis, 2 ventricular extrasystole, 2 myocardial infarction, 1 post Covid infection).

### Cardiovascular magnetic resonance imaging

CMR Imaging was performed using a multi-transmit 3.0 Tesla magnetic resonance imaging system (Achieva, Philips Healthcare, Best, The Netherlands; Release 5.6.1, respectively) with dStream technology. All volunteers were examined in supine position. In order to enable cardiac-triggering acquisitions, a vector electrocardiogram was applied. The maximum gradient performance was 40 mT/m, slew rate = 200 mT/m/ms and signal reception was achieved using a cardiac phased-array coil. All patients were examined according to the appropriate standard procedures intended for investigation of their respective cardiovascular disease, including the typical standard 2-chamber and 4-chamber views aligned along the long-axis of the left ventricle. To assess ventricular cardiac function, morphology and strain, cine steady-state free-precession acquisitions (TR/TE/flip angle = 2.7 ms/1.35 ms/42°) were acquired with 30 reconstructed cardiac phases per cardiac cycle keeping breath-holding periods <12 s. Spatial resolution was 1.5 mm × 1.5 mm × 8 mm.

**Figure 1:**
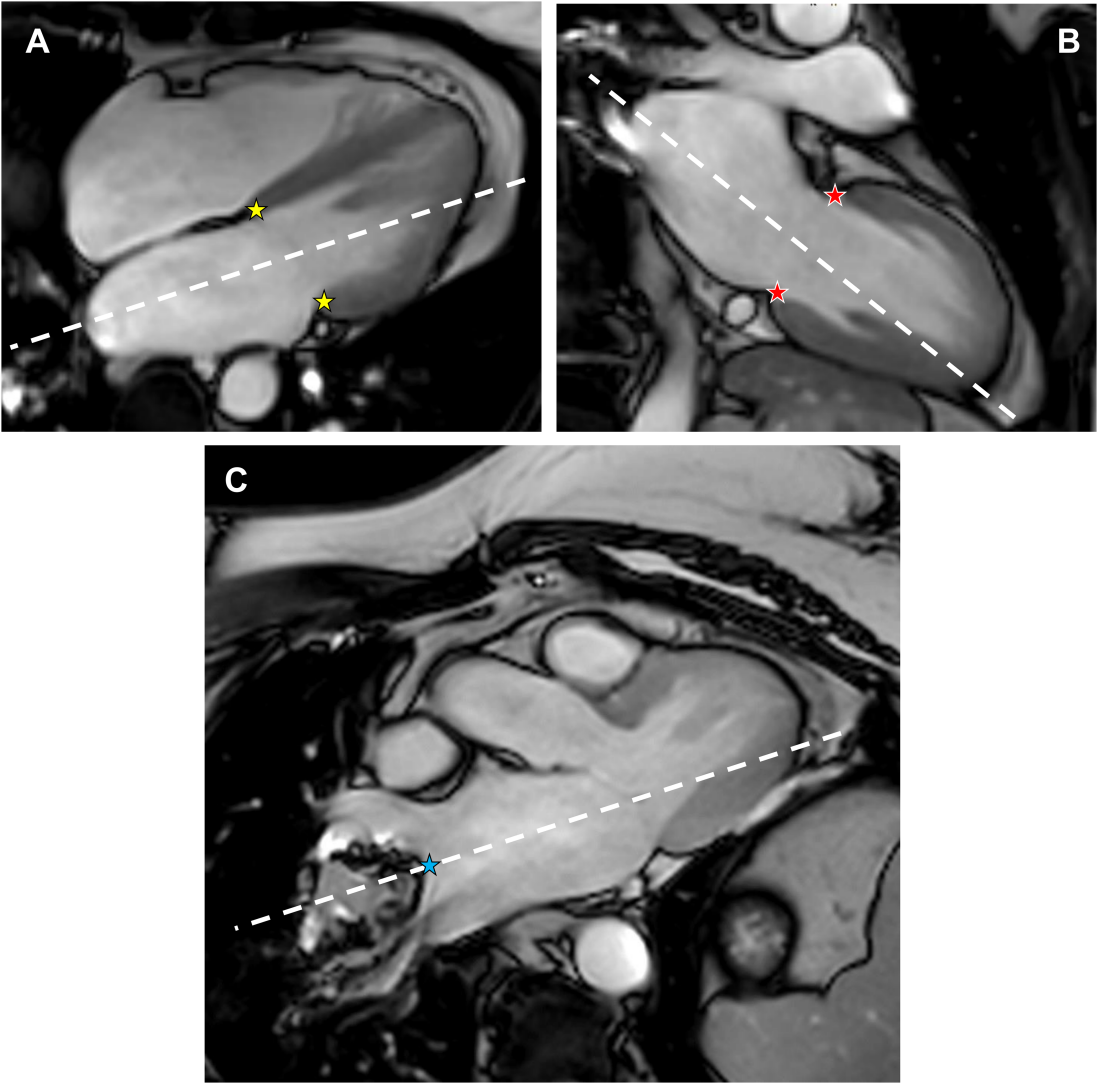
Optimized planning along the main axes of the left atrium to assess volume, strain and strain rates. **(A)** Standard cine SSFP 4-chamber view at end-systole. **(B)** Standard cine SSFP 2-chamber view at end-systole. **(C)** Additional end-systolic cine SSFP view depicting the mitral valve and both the right upper pulmonary vein and the right lower pulmonary vein, which represents the longest extent of the left atrium. The three-point planning tool was used to define the optimized 4-chamber view (yellow stars and the blue star) and the optimized 2-chamber view (red stars and the blue star).

The determination of left atrial volumes and left atrial strain are obtained by two different planning procedures. (a) *Standard LA planning*: The traditional standard 2-chamber and 4-chamber views are used, which are aligned along the long-axis of the left ventricle. (b) *Optimized LA planning*: An additional planning scan is required to optimally determine left atrial (LA) volumes and LA cardiac strain. For example, this survey scan can be obtained using a three-point planning tool installed on the scanner's platform, where the first point is placed in the right upper pulmonary vein (RUPV), the second point in the right lower pulmonary vein (RLPV), and the third point in the center of the mitral value of the 2-chamber or 4-chamber view (Figure 1C). Note: Systolic heart frames should be used for planning, as the LA dimensions are largest in systole. Preferably, a previously acquired axial cine stack that includes all pulmonary veins can be used for this purpose, however axially acquired survey scans may also be acceptable.

For the optimized planning procedure, the 2-chamber and 4-chamber views, which were previously aligned to the left ventricle, are copied, but aligned along the main axis of the LA, as shown in Figure 1. To capture the LA main axis of the optimized “4-chamber LA view”, the three-point planning tool is used again, with the first two points set to the septal and lateral intersection of a standard 4-chamber systolic view, respectively, and the third point set to the minimum between the RUPV and the RLPV of the additional planning survey using a systolic heart frame as well. Accordingly, the optimized “2-chamber LA view” is obtained by placing the first two points on the anterior and posterior intersections of a standard 2-chamber systolic view and the third point on the minimum between the RUPV and the RLPV in a systolic heart frame of the additional planning survey.

**Figure 2:**
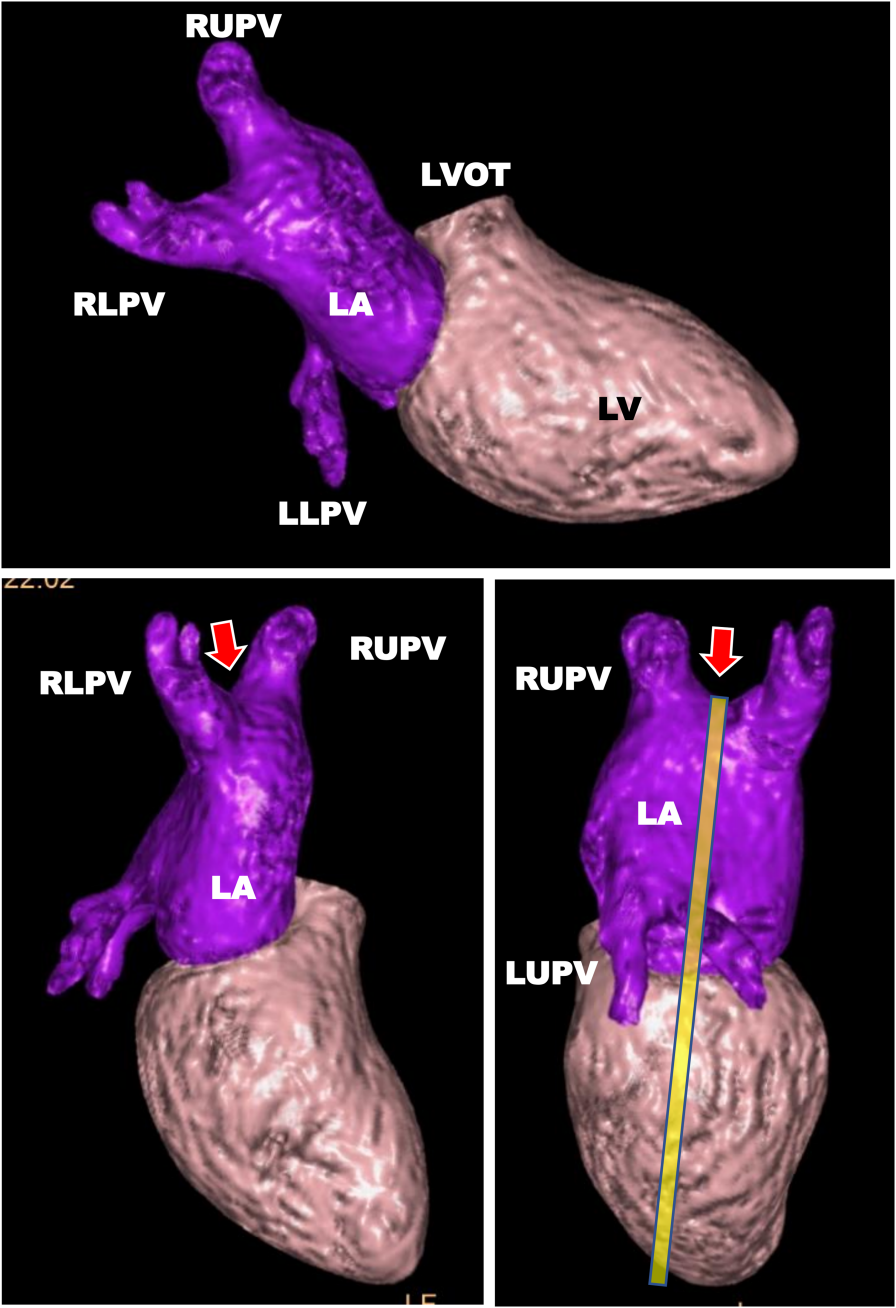
Illustration of the most distant position (arrow), typically best represented by the valley between the right upper pulmonary vein and the right lower pulmonary vein, to visualize the main axis of the left atrium using a 3D model. Heart segmentation including surface rendering using the whole heart analysis software IntelliSpace Portal (Version 12.1, Philips Medical Systems, Best, The Netherlands). For the reason of simplicity, only the left ventricle (LV) and the left atrium (LA) are shown to illustrate the spatial position of the two chambers. RLPV, right lower pulmonary vein; RUPV, right upper pulmonary vein; LLPV, left lower pulmonary vein; LUPV, left upper pulmonary vein.

Note: The minimum between the RUPV and the RLPV is the preferred position for defining the main axis of the LA, as this position usually represents the largest extent of the LA, as illustrated in Figure 2 and Supplementary Material S1.

### Left atrial volume analysis

The volume analysis of the left atrium was performed with the biplanar LAX module of the CVI42® software package (Circle Cardiovascular Imaging Inc., Calgary, Canada, Release 6.0.2). After loading the appropriate 2-chamber and 4-chamber views (standard LA planning or optimized LA planning), the contour detection of the left atrium in the end-systolic and end-diastolic heart frame was performed using the AI option version only for the current image (phase). If needed, a manual adaption of the contours was done to calculate both the indexed left atrial maximum volume (LA-Vmax_i_) and the indexed left atrial minimum volume (LA-Vmin_i_). To determine the indexed volume before left atrial contraction (LA-Vpre_i_), the same AI option was applied to a diastolic heart frame representing the maximium volume prior left atrial contraction. Note: This heart frame is first defined in the strain analysis module (see below). After calculation of the strain values, the software displays—among other parameters—the volume-to-time curve, from which the position of the heart frame prior left atrial contraction can be derived.

### Left atrial strain analysis

The left atrial strain analysis was conducted using the left ventricular strain module of the CVI42® software package. For the sake of simplicity, the LA longitudinal strain was expressed in positive values.

**Figure 3:**
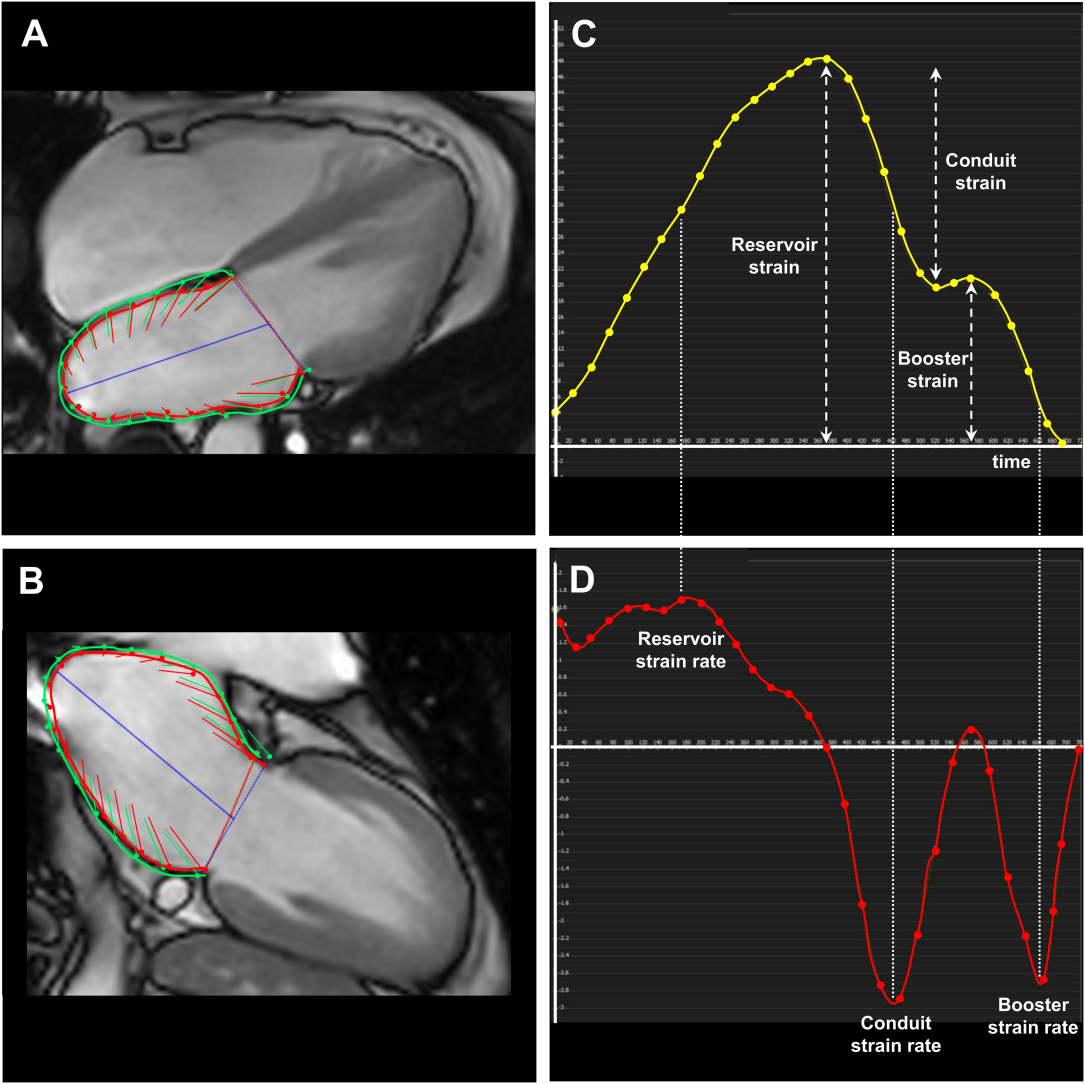
Strain analysis applying the optimized planning procedure to correctly evaluate the strain, strain rate as well as the left atrial volumes. **(A,B)** Strain illustration by boundary points in a 4-chamber and 2-chamber view, respectively, in a 53-year-old women with sarcoidosis. Accordingly, **(C)** the strain-to-time curve and **(D)** the corresponding strain rate-to-time curve are displayed.

In the first step, the respective 2-chamber and 4-chamber views (standard LA planning or optimized LA planning) with the end-systolic and end-diastolic chamber contours, which were previously defined in the biplanar LAX module, were uploaded to the strain module. With the exception of the LA contours, all other contours have been removed. As the left ventricular strain module was exceptionally used to calculate LA strain, new “left ventricular endocardial contours” had to be drawn exactly on the LA contours previously defined in the biplanar LAX module. In the subsequent step, the “old” LA contour should be deleted. Additionally, the epicardial contours were delineated around the endocardial contours (Figure 3). Afterwards, the strain analysis was started. However, the calculated LA strain values are now available under the left ventricular tabs. In order to represent LA longitudinal strain as positive values, a manual definition of the end-diastolic heart frame, which corresponds to LA-Vmin_i_, and of the end-diastolic heart frame, which corresponds to LA-Vmax_i_, had to be done. The global longitudinal reservoir LA strain (GLS-LA_res_), the global longitudinal conduit LA strain (GLS-LA_con_) and the global longitudinal booster LA strain (GLS-LA_boo_) as well as the corresponding parameters for the strain rates (GLSR-LA_res_, GLSR-LA_con_, GLSR-LA_boo_) were taken from the corresponding time curves or the report print-outs. The values for the long axis displacement were taken from the scientific data report.

In the next step, the appropriate heart frame for the calculation of the LA-Vpre_i_ (LA volume prior left atrial contraction, see above) could be determined in the endocardial volume-to-time curve, whereby the second maximum LA volume, which occurs in late diastole, marks the appropriate position. The LA volume of this heart frame should be used to calculate LA-Vpre_i_ in the biplanar LAX module (see above).

### Statistical analysis

Statistical analysis was carried out utilizing IBM SPSS Statistics (version 29.0.0.0, IBM Deutschland GmbH). Normal distribution was tested using Shapiro–Wilk test. Continuous variables were presented as mean ± standard deviation (SD) when normally distributed, otherwise as median with interquartile range. Differences in continuous variables between the two different planning procedures for assessment of the left atrial strain were evaluated using a paired Student's *t*-test for normal distribution and the Wilcoxon test for non-normal distribution. Furthermore, the Bland-Altman statistic was applied to elucidate the agreement between the two planning strategies. Inter-observer and intra-observer variability was tested by Bland-Altmann analysis and the intra-class-correlation coefficients (ICC, two-way random effects, consistency, respectively, two-way mixed model, absolute agreement (11). A *P* value <0.05 was considered statistically significant.

## Results

Twenty-one patients with different cardiovascular diseases were enrolled in this study with a mean age of 48.3 ± 15.2 years (11 men, Table 1).

**Table 1:** Patient baseline characteristics (*N* = 21).

Parameter	All
Male/female	11/10
Age (years)	48.3 ± 15.2
Weight (kg)	75.5 ± 15.3
Height (cm)	180 {162; 181}
Body surface area (m^2^)	1.89 ± 0.24
Body mass index (kg/m^2^)	25.2 ± 3.9

Data reported as mean ± standard deviation or median (interquartile range).

When using the optimized planning protocol, all indexed left atrial volumes were statistically significantly increased by approximately 25% than when using the standard planning protocol (*P* < 0.001, Table 2). In contrast, the reservoir left atrial global longitudinal strain GLS-LA_res_ was statistically significantly lower applying the optimized planning protocol compared to the standard planning protocol (27.3 ± 9.5% vs. 37.1 ± 10.8%, *P* < 0.001), as was the conduit left atrial global longitudinal strain GLS-LA_con_ (17.1 ± 6.4% vs. 24.7 ± 9.8%, *P* < 0.001) and the booster left atrial global longitudinal strain GLS-LA_boo_ (11.7 ± 4.5% vs. 14.5 ± 4.7%, *P* < 0.001). Likewise, all corresponding left atrial global longitudinal strain rates were found reduced using the optimized planning protocol with *P*-values <0.007.

**Table 2:** Planning-related differences in left atrial volumes, strain and strain rate.

Parameter	Standard LA planning	Optimized LA planning	*P*-value	Bland Altman statistics mean difference (%) [limits of agreement]
LA-Vmax_i_ (ml/m^2^)	33.2 ± 9.7	42.6 {35.6; 52.3}	**<0.001** *	−12.6 [−34.8 to 9.7]
LA-Vmin_i_ (ml/m^2^)	13.4 {10.9; −17.0}	19.8 {14.7; 27.0}	**<0.001** *	−7.3 [−23.8 to 9.1]
LA-Vpre_i_ (ml/m^2^)	23.3 ± 9.0	31.0 {21.3; 37.5}	**<0.001** *	−9.6 [−31.4 to 12.1]
LA-EF_tot_ (%)	56 ± 12	53 ± 11	0.155	2.9 [−14.6 to 20.4]
LA-EF_pas_ (%)	31 ± 13	30 ± 13	0.712	1.0 [−22.6 to 24.5]
LA-EF_act_ (%)	36 ± 11	32 ± 12	**0.034**	3.9 [−11.6 to 19.5]
GLS-LA_res_ (%)	37.1 ± 10.8	27.3 ± 9.5	**<0.001**	9.8 [−9.5 to 29.0]
GLS-LA_con_ (%)	24.7 ± 9.8	17.1 ± 6.4	**<0.001**	7.6 [−7.3 to 22.6]
GLS-LA_boo_ (%)	14.5 ± 4.7	11.7 ± 4.5	**<0.001** *	2.8 [−2.4 to 8.0]
GLSR-LA_res_ (s^−1^)	1.71 ± 0.44	1.50 {0.95; 1.60}	**0.007** *	0.3 [−1.3 to 1.8]
GLSR-LA_con_ (s^−1^)	−1.87 ± 0.71	−1.34 ± 0.54	**<0.001**	−0.5 [−1.5 to 0.5]
GLSR-LA_boo_ (s^−1^)	−1.93 ± 0.61	−1.65 ± 0.64	**<0.001**	−0.3 [−1.0 to 0.4]
Long axis displacement (mm)	13.7 ± 3.5	12.0 ± 2.8	**0.023**	1.7 [−4.6 to 8.0]

Data reported as mean ± standard deviation or median {interquartile range}. ICC, intra-class correlation coefficient; EF_tot_, total emptying fraction; EF_pas_, passive emptying fraction; EF_act_, active emptying fraction; GLS, global longitudinal strain; LA_res_, reservoir left atrial; LA_con_, conduit left atrial; LA_boo_, booster left atrial; Vmax_i_, indexed maximum volume; Vmin_i_, indexed minimum volume; Vpre_i_, indexed volume before atrial contraction.

* Wilcoxon-test, otherwise paired Student's *t*-test.

Significant values are shown in bold.

Regardless of the planning procedure, the intrarater reliability assessed with the Bland-Altman statistics was low, indicating almost no bias and small limits-of-agreement (Table 3). The mean differences of indexed left atrial volumes were less than 1 ml/m^2^, the left atrial ejection fractions below 3% and the left atrial global longitudinal strains remained <2.5%. All ICCs were >0.8 except for GLS-LA_res_ (0.783, standard planning) and the left atrial passive emptying fraction LA-EF_pas_ (0.641, optimized planning). A somewhat lower agreement was found for interrater reliability. The mean differences of indexed left atrial volumes were less than 1.5 ml/m^2^, the left atrial ejection fractions below 3% and the left atrial global longitudinal strains remained ≤6%. The ICCs were slightly lower compared to the intrarater statistics.

**Table 3:** Intra- and interrater reliability by Bland-Altman statistics and intra-class correlation in global left atrial strain (*N* = 10).

Parameter	Standard LA planning			Optimized LA planning		
	Mean difference [limits of agreement]		ICC	Mean difference [limits of agreement]		ICC
Intrarater						
LA-Vmax_i_ (ml/m^2^)	−0.6	[−6.0 to 4.8]	0.958	−0.7	[−5.3 to 3.9]	0.984
LA-Vmin_i_ (ml/m^2^)	−0.2	[−1.2 to 0.9]	0.997	−0.1	[−2.9 to 2.7]	0.993
LA-Vpre_i_ (ml/m^2^)	0.0	[−1.4 to 1.5]	0.997	0.6	[−4.9 to 6.1]	0.976
LA-EF_tot_ (%)	−0.6	[−10.3 to 9.0]	0.915	−0.9	[−9.7 to 8.0]	0.913
LA-EF_pas_ (%)	−1.5	[−14.3 to 11.4]	0.847	−2.8	[−19.5 to 14.0]	0.641
LA-EF_act_ (%)	0.9	[−4.7 to 6.5]	0.959	1.9	[−6.5 to 10.4]	0.906
GLS-LA_res_ (%)	−0.1	[−12.9 to 12.7]	0.783	2.2	[−6.0 to 10.4]	0.852
GLS-LA_con_ (%)	0.2	[−8.5 to 9.0]	0.865	1.4	[−5.5 to 8.3]	0.819
GLS-LA_boo_ (%)	0.4	[−3.1 to 3.9]	0.922	0.1	[−2.2 to 2.4]	0.957
Interrater						
LA-Vmax_i_ (ml/m^2^)	−0.4	[−1.5 to 0.7]	0.967	−1.3	[−3.6 to 1.0]	0.989
LA-Vmin_i_ (ml/m^2^)	0.1	[−0.5 to 0.7]	0.996	−0.9	[−3.4 to 1.7]	0.985
LA-Vpre_i_ (ml/m^2^)	0.4	[−1.3 to 2.1]	0.993	−1.0	[−3.7 to 1.6]	0.964
LA-EF_tot_ (%)	−1.0	[−4.6 to 2.5]	0.936	0.5	[−4.2 to 5.1]	0.858
LA-EF_pas_ (%)	−2.0	[−7.2 to 3.3]	0.765	0.0	[−6.3 to 6.2]	0.618
LA-EF_act_ (%)	0.3	[−4.1 to 4.8]	0.908	0.8	[−7.0 to 8.7]	0.950
GLS-LA_res_ (%)	−6.0	[−24.5 to 12.5]	0.874	−5.0	[−17.9 to 7.9]	0.741
GLS-LA_con_ (%)	−2.5	[−14.8 to 9.8]	0.872	−1.3	[−9.1 to 6.6]	0.883
GLS-LA_boo_ (%)	−2.7	[−9.9 to 4.5]	0.855	−3.3	[−9.8 to 3.2]	0.775

ICC, intra-class correlation coefficient; EF_tot_, total emptying fraction; EF_pas_, passive emptying fraction; EF_act_, active emptying fraction; GLS, global longitudinal strain; LA_res_, reservoir left atrial; LA_con_, conduit left atrial; LA_boo_, booster left atrial; Vmax_i_, indexed maximum volume; Vmin_i_, indexed minimum volume; Vpre_i_, indexed volume before atrial contraction.

## Discussion

LA volume is a key biomarker in heart failure (4) and cardiac amyloidosis (2), predicting atrial fibrillation (3), heart failure (4), and mortality (5). Its remodeling reflects alterations volume and pressure, making precise quantification essential for assessing disease progression and treatment efficacy. The growing use of CMR to evaluate left atrial strain and volumetrics as clinical biomarkers for disease diagnosis and prognosis underscores the importance of obtaining accurate and representative values. The majority of CMR literature adapts a standard planning technique that is aligned to the axes of the left ventricle when acquiring LA volumetrics and strain. The present study demonstrates the quantitative discrepancies between a standard and optimized planning procedure of the LA with the following novel findings:

1. Left atrial volumetrics are significantly increased using optimized vs. standard planning conditions.
2. Left atrial global reservoir, conduit and booster strain and strain rate is significantly reduced using optimized vs. standard planning conditions.
3. Independent of the optimized or standard planning procedure, low levels of intra- and interrater variations were observed.

There is limited consensus in the literature regarding the quantification of left atrial volume (12). Despite this, the biplane area-length method based on 2- and 4-chamber views remains a widely used approach (9, 10, 13, 14). However, to the best of our knowledge, optimized planning protocols for LA have not been described in CMR literature. In retrospect, the extensive data on LA volumetrics and strain collected for both healthy and pathological states may reflect a systematic error that has been perpetuated over time. While the clinical interpretations based on this data most likely maintain their meaning, since the significant deviations are likely measurable across both planning methods, they do not accurately represent true physiological values.

Literature on intermodal LA volumetric analyses remains very limited. Echocardiographic measurements have shown systematic underestimations of LA volume (15) and LA strains (16) in contrast to CMR quantifications. These findings underline that LA volumes are not interchangeable between echocardiography and CMR. Regarding volumetric analysis using computed tomography (CT), studies have reported higher absolute LA volume measurements compared to CMR, though these differences have not reached statistical significance (17). While this trend may partly explain the differences observed in the present study between standard LV-based planning and optimized LA-based planning, intermodality studies generally indicate substantial variability across imaging techniques. This variability underscores the necessity of careful methodological considerations when assessing LA size and function, reinforcing the clinical importance of precise chamber alignment for accurate and reproducible measurements.

As already visually apparent, the optimized LA planning procedure captures the longest axes more accurately than the standard planning procedure. As a result, the percentage difference between end-systolic and end-diastolic dimensions is significantly smaller, leading to lower strain values compared to the standard planning procedure.

To illustrate this with an example:

• Using the standard planning method with the 2-chamber view, the difference in LA dimensions is 5 cm in end-systole and 2.5 cm in end-diastole. This results in a strain calculation of (5–2.5)/5 = 0.5, or 50%.
• With the optimized planning method in the 2-chamber view, the difference in LA dimensions is 10 cm in end-systole and 7.5 cm in end-diastole, assuming the LA expands by the same 2.5 cm. In this case, the strain is (10–7.5)/10 = 0.25, or 25%.

**Figure 4:**
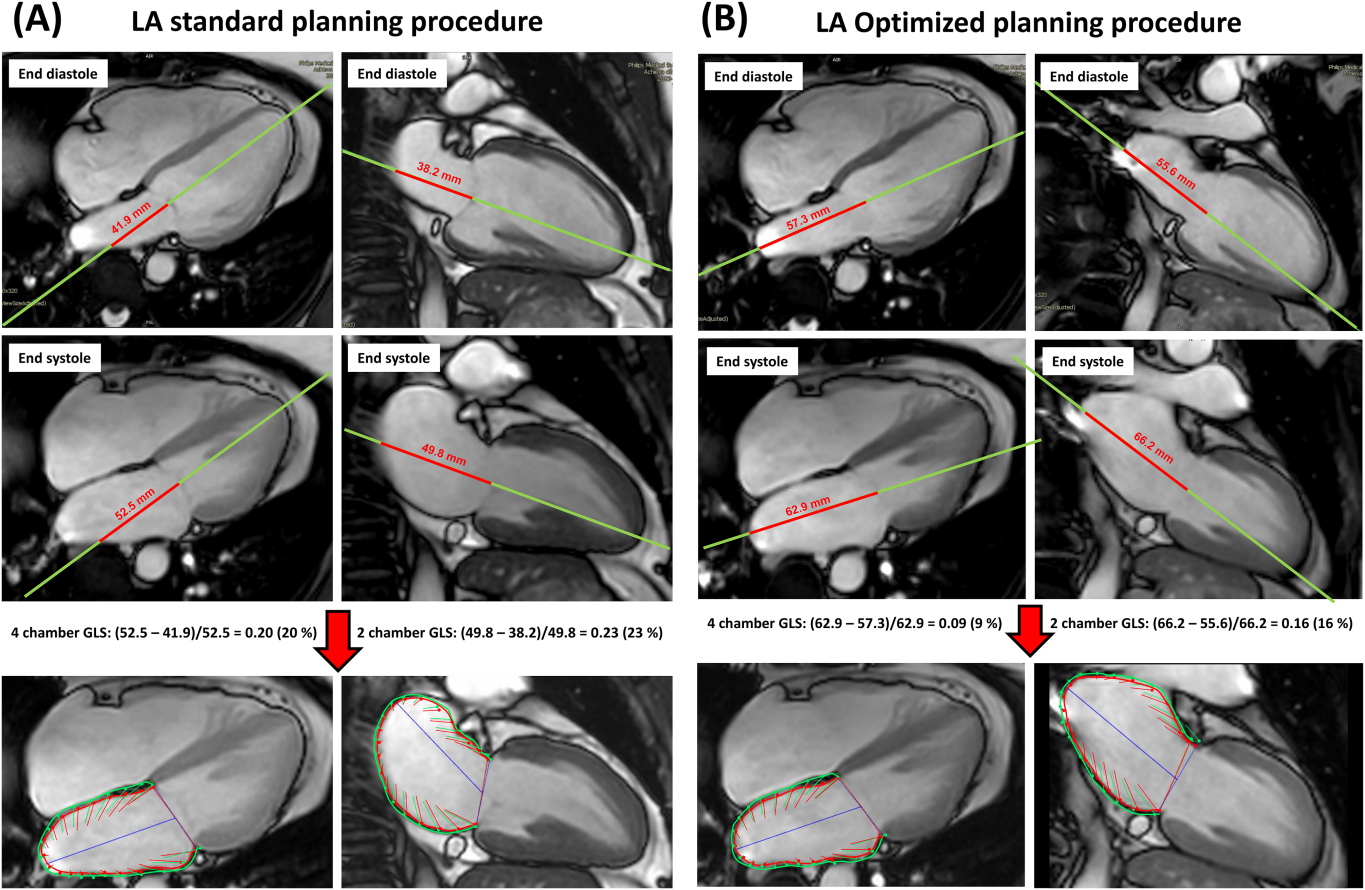
**(A,B)** Left atrial strain evaluation by standard planning or optimized planning. In case of standard planning (2-chamber and 4-chamber view aligned along the main axis of the left ventricle), the longest extent of the left atrium is usually not correctly captured (see red line), resulting in overestimations of strain and in underestimations of volumes. In contrast, by applying the optimized planning procedure, a correct definition of the longest LA extent is achieved, which improves the accuracy of left atrial strain, strain rate and volume values.

As supported by the present study findings, the observed discrepancies between both planning methods are of technical origin. Utilizing the standard planning protocol, dislocates the centerline and thereby cuts the LA at an angle (Figure 4A,B, Supplementary Material S1). Throughout a cardiac cycle, the quantified deformation and wall motion between end-systole and end-diastole alter significantly due to this angled cut, resulting in decreased volumetrics with larger strain values. In contrast, adapting the optimized LA planning procedure, centers the axis lines with respect to the right upper and right lower pulmonary vein and the central point of the mitral valve, thereby capturing more accurate chamber dimensions. Consequently, the quantified LA volumetrics are larger compared to values derived from the standard protocol, with lowered cardiac wall deformation reflected in the strain and strain rates. Moreover, these observations may explain the significant intermodal measurement differences recently reported (16), where CMR showed significantly higher reservoir and conduit strains compared to transthoracic echocardiography.

## Limitations

This study has several limitations that must be acknowledged. The cohort size is small, necessitating larger studies to consolidate its findings. The proposed optimized planning procedure for the “new” LA 2-chamber cine scan occasionally includes portions of the left ventricular outflow tract, which could affect the accuracy of LA strain measurements. Although this procedure aims to capture the longest LA dimensions by using the central position between the right upper and right lower pulmonary veins, it may be beneficial to use the contralateral position of the right upper pulmonary vein. However, this alternative positioning was not validated in this small study. Additionally, the study did not compare CMR with echocardiography data, which could further support the adoption of the optimized planning protocol.

## Conclusion

Left atrial volumes, strain and strain rates strongly depend on the CMR planning procedure. In contrast to the typically applied standard planning, the optimized left atrial planning yields higher maximal volumes, minimal volumes and volumes before atrial contraction as well as lower left atrial reservoir, conduit and booster global longitudinal strains and strain rates. An optimized planning procedure is proposed and recommended to improve the reliability of left atrial functional parameters.

## Data Availability

The raw data supporting the conclusions of this article will be made available by the authors, without undue reservation.

## Supplementary material

Supplementary Material S1 To better visualize the positional relationship of the left ventricle and left atrium, a coronal navigator- and vector-ECG-triggered three-dimensional non-selective whole-heart acquisition with a compressed SENSE reduction factor of 6, acquisition window in end-diastole, 300 slices, acquisition time 3:09 min was applied. Surface rendering of the left ventricle and left atrium was performed using the Philips IntelliSpace Portal platform. Data were exported in binary STL format and transferred to a Philips iSUITE workstation. To illustrate the misregistration that occurs when using the standard planning technique, which is used to accurately visualize the left ventricle but not the left atrium, the typical 2-chamber, 4-chamber and short-axis views are shown in the video.
